# Advances in Therapeutics Research for Demyelinating Diseases

**DOI:** 10.3390/ph18121835

**Published:** 2025-12-01

**Authors:** Jinhui Jiang, Yuchen Sun, Yuan Ma, Chenhui Xu, Xiaofeng Zhao, Hui Fu

**Affiliations:** 1 Zhejiang Key Laboratory of Organ Development and Regeneration, College of Life and Environmental Sciences, Hangzhou Normal University, Hangzhou 311121, China; 2024111010032@stu.hznu.edu.cn (J.J.); 2024111010031@stu.hznu.edu.cn (Y.S.); 2 Department of Anatomy and Embryology, School of Basic Medical Sciences, Hangzhou Normal University, Hangzhou 311121, China; mayuan2019@126.com (Y.M.); 13732366277@163.com (C.X.)

**Keywords:** demyelinating diseases, therapeutics research, multiple sclerosis, remyelination, neuroinflammation, epigenetic therapy

## Abstract

Demyelinating diseases comprise a group of chronic and debilitating neurological disorders, with the destruction of the myelin sheath serving as the core pathological hallmark. The central pathogenesis involves immune-mediated damage to oligodendrocytes (Ols) and myelin breakdown, accompanied by a vicious cycle of neuroinflammation and impaired epigenetic repair. Current therapeutic strategies, including conventional immunomodulatory agents to targeted monoclonal antibodies, effectively control disease relapses but exhibit limited efficacy in promoting neural repair. Consequently, research focus is increasingly shifting towards neuroprotective and remyelination strategies. In this context, Emerging therapeutic promise stems primarily from two fronts: the advent of novel pharmaceuticals, such as remyelination-promoting drugs targeting oligodendrocyte maturation, interventions inhibiting epigenetic silencing, signal pathway inhibitors, and natural products derived from traditional Chinese medicine; the development of innovative technologies, including cell therapies, gene therapy, exosome and nanoparticle-based drug delivery systems, as well as extracellular protein degradation platforms. Nevertheless, drug development still faces challenges such as disease heterogeneity, limited blood–brain barrier penetration, long-term safety, and difficulties in translating findings from preclinical models. Future efforts should emphasize precision medicine, multi-target synergistic therapies, and the development of intelligent delivery systems, with the ultimate goal of achieving a paradigm shift from delaying disability progression to functional neural reconstruction.

## 1. Introduction

Demyelinating diseases constitute a class of neurological disorders characterized by damage to myelin as the primary pathological feature. Based on the site of pathology, they are classified into two major categories: central nervous system (CNS) demyelinating diseases (e.g., multiple sclerosis [MS] [1,2] and neuromyelitis optica spectrum disorder [NMOSD]) and peripheral nervous system (PNS) demyelinating diseases (e.g., Guillain–Barré syndrome (GBS) and chronic inflammatory demyelinating polyneuropathy (CIDP) [3]).

The core pathogenic mechanism of demyelinating diseases involves autoimmune response-mediated targeted injury to myelin [4]. Environmental factors (such as Epstein–Barr virus [EBV] infection [5]) activate myelin-specific T cells via molecular mimicry, leading to a breakdown of immune tolerance in genetically susceptible individuals (e.g., carriers of the *HLA-DRB1* allele) [6,7]. Activated CD4^+^ T cells (particularly the Th17 subset [8]) cross the blood–brain barrier (BBB), releasing pro-inflammatory cytokines (IL-6 and IL-17) and recruiting B cells to produce anti-myelin antibodies (e.g., anti-MOG and anti-AQP4). These antibodies directly damage oligodendrocytes through complement-dependent cytotoxicity (CDC) and antibody-dependent cellular phagocytosis (ADCP), resulting in myelin breakdown. Concurrently, a vicious cycle arises from glial–immune cell interactions: the caspase-3-dependent cleavage of GSDME protein in microglia triggers pyroptosis, releasing pro-inflammatory cytokines such as IL-1β and IL-18, which thereby amplify neuroinflammation and inhibit the autophagic clearance of myelin debris [9]. Furthermore, Wnt-signal-hyperactivated oligodendrocyte precursor cells (OPCs) secrete the chemokine CCL4, recruiting CD4^+^ T cells and inducing macrophages to transform into cytotoxic subsets with high TNFα expression, which directly kill OPCs and exacerbate demyelination [8]. Additionally, BBB disruption introduces peripheral metabolic toxins, causing secondary injury. Leaked low-density lipoproteins (LDL) activate glycolytic reprogramming in microglia, resulting in excessive phagocytosis of myelin debris and bursts of reactive oxygen species (ROS). Notably, the chronic inflammatory microenvironment ultimately blocks repair processes through epigenetic silencing mechanisms: DNMT1-mediated DNA hypermethylation and H3K27me3 histone modifications repress key myelin genes (e.g., myelin basic protein (MBP) and proteolipid protein (PLP)). Conversely, targeted inhibition of histone acetyltransferases (HATs) can upregulate interleukin-33 (IL-33) to restore oligodendrocyte precursor cell (OPC) differentiation capacity [10], offering a novel avenue for regenerative intervention.

Using multiple sclerosis (MS) as an example [11,12,13], epidemiological studies indicate a global prevalence of approximately 35.9 per 100,000 people, varying from 3–5 per 100,000 in low-risk regions (e.g., Asia) to 300 per 100,000 in high-risk areas (e.g., Northern Europe and North America), forming a distinct latitudinal gradient (higher risk at higher latitudes). The incidence in females is 2–3 times that in males. In recent years, urbanization (studies in Italy showed a 29% higher risk in urban residents) and air pollution (PM2.5 promoting neuroinflammation) have emerged as significant risk factors, thereby highlighting the interplay between environmental and genetic factors in the pathogenesis of demyelinating diseases.

As a leading cause of disability due to neurological dysfunction in young adults, demyelinating diseases impose a substantial burden on healthcare systems. The resulting irreversible neurological damage—including paralysis, blindness, severe pain, and cognitive decline—not only undermines employability but is also associated with elevated depression rates and disruption of family caregiving structures. Current management faces a triple challenge: diagnostic delays, limited drug accessibility, and the inefficacy of conventional therapies against disease progression. Although existing treatments (e.g., high-dose corticosteroid pulses [14] and immunosuppressants) can partially control acute inflammatory responses, they exhibit limited efficacy against progressive neurodegeneration and remyelination. Long-term use increases the risk of severe adverse effects, including infections and hepatorenal toxicity. Consequently, developing novel therapeutics that combine potent remyelination capacity with long-term safety represents a critical unmet need in contemporary neuroscience [15].

**Figure 1:**
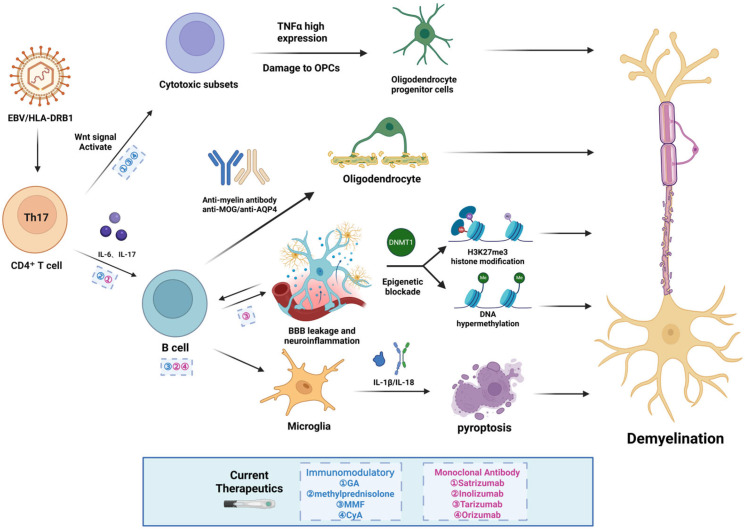
**Core pathophysiology of demyelinating diseases and representative drug targets.** This figure outlines the pathogenic cascade from initial autoimmune attack to failed repair, highlighting key signaling nodes and the corresponding therapeutic strategies at distinct pathological tiers. (1) Initiation: In genetically susceptible individuals, triggers like Epstein–Barr virus (EBV) activate myelin-specific CD4^+^ T cells (e.g., Th17), a key node, leading to a breakdown of immune tolerance. (2) Amplification and Damage: Activated T cells breach the blood–brain barrier (BBB), a key node, recruiting B cells and cytotoxic subsets. Anti-myelin antibodies (e.g., anti-MOG/AQP4) directly damage oligodendrocytes, causing demyelination. (3) Vicious Cycle: Microglial pyroptosis (a key node via *GSDME*) releases pro-inflammatory cytokines (IL-1β, IL-18), further amplifying neuroinflammation and inhibiting repair. (4) Repair Failure: The chronic inflammatory milieu induces an epigenetic blockade (a key node via H3K27me3/DNA methylation), repressing myelin genes and arresting oligodendrocyte precursor cell differentiation. The sites of action for representative approved therapeutics (e.g., Natalizumab, Rituximab, Eculizumab) are indicated at their respective pathological nodes, illustrating that current strategies primarily target immunomodulation and inflammatory control. However, significant challenges remain in overcoming disease heterogeneity, bypassing the BBB delivery barrier, and achieving effective remyelination.

The core pathophysiology of demyelinating diseases, spanning from initial autoimmune attack to the failure of repair, along with the sites of action for representative approved therapeutics, is summarized in Figure 1.

## 2. Current Landscape of Therapeutics Research for Demyelinating Diseases

**Figure 2:**
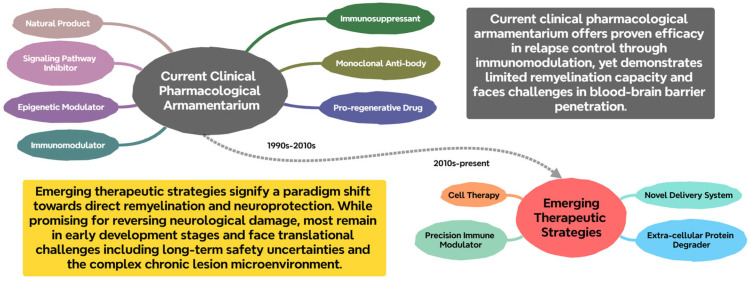
**Therapeutic landscape and strategic evolution in demyelinating diseases.** The diagram categorizes the existing pharmacological armamentarium, primarily focused on immunomodulation for relapse control, and contrasts it with the strategic pivot towards emerging therapies that target remyelination and neuroprotection. Key challenges for each approach are highlighted, including the limited reparative capacity and blood–brain barrier (BBB) delivery constraints of established drugs, alongside the translational hurdles of long-term safety and efficacy validation for novel strategies. This schematic provides an integrated overview of the field’s transition from disease modification to the pursuit of functional reconstruction.

To provide a conceptual overview of the therapeutic advances and strategic shift from immunomodulation to CNS repair, a summary of the current pharmacological armamentarium and emerging strategies is presented in Figure 2.

### 2.1. Immunomodulatory and Immunosuppressive Agents

#### 2.1.1. Immunomodulators

Interferon-beta (IFN-β) and glatiramer acetate (GA), serving as first-line immunomodulatory agents for multiple sclerosis (MS), regulate the disease process through distinct mechanisms. IFN-β primarily alleviates central nervous system inflammation by inhibiting T-cell migration across the blood–brain barrier, downregulating pro-inflammatory cytokines (e.g., IFN-γ), and enhancing regulatory T-cell function. GA, a representative glatiramer, is a synthetic polypeptide mixture composed of four amino acids with a more diversified mechanism of action: first, it competitively binds to MHC-II molecules on antigen-presenting cells (APCs), mimicking myelin basic protein (MBP) to induce immune tolerance; second, it promotes the differentiation of anti-inflammatory T helper 2 (Th2) cells, which secrete cytokines such as IL-4 and IL-10; third, it activates regulatory T cells (Tregs) and induces a “bystander suppression” effect, thereby locally suppressing inflammation at lesion sites [15,16]. Recent studies have further revealed that GA directly acts on APCs and B cells, enhancing the secretion of neurotrophic factors (e.g., BDNF), thus concurrently exerting neuroprotective effects.

In clinical practice, both agents are indicated for relapsing-remitting MS (RRMS) [17] and clinically isolated syndrome (CIS). Large controlled trials (e.g., PreCISe) confirmed that the GA group exhibited a substantially lower risk of converting to clinically definite MS (CDMS) compared to the placebo group, with GA delaying the time to conversion to definite MS by 386 days (corresponding to a 45% risk reduction) [18]. Head-to-head studies (BEYOND [19], REGARD [20], BECOME [21]) demonstrated similar efficacy between GA and IFN-β in reducing relapse rates and disability progression, although GA offers an advantage in terms of cost-effectiveness. Studies of combination therapy (e.g., the GLANCE trial combining GA with natalizumab) demonstrated superior efficacy in suppressing radiological activity (e.g., new lesion formation) compared to GA monotherapy, with good short-term tolerability [22]. Furthermore, patients receiving induction therapy with GA combined with mitoxantrone showed greater improvement in Expanded Disability Status Scale (EDSS) scores and a more marked reduction in the volume of T1-weighted hypointense lesions (“black holes”), suggesting that combination regimens could potentially mitigate irreversible neurological damage [23]. However, neither agent is effective against primary progressive MS (PPMS), highlighting the limitations imposed by disease heterogeneity.

In addition to the injectable immunomodulators mentioned above, sphingosine-1-phosphate (S1P) receptor modulators, as an important class of oral disease-modifying therapies (DMTs), have further expanded the treatment options for relapsing forms of multiple sclerosis (MS). The mechanism of action of S1P receptor modulators is primarily based on the modulation of S1P receptors, which belong to the G protein-coupled receptor family and are widely expressed on immune cells, within the central nervous system, cardiovascular system, and other tissues. By binding with high affinity to S1P receptor subtypes (e.g., S1P_1_, S1P_3_, S1P_4_, S1P_5_), these agents block lymphocyte egress from lymph nodes, leading to a reduction in peripheral blood lymphocyte counts. This consequently decreases lymphocyte infiltration into the central nervous system, alleviating inflammatory responses and demyelinating injury. Moreover, in preclinical models, the S1P signaling pathway has been associated with increased oligodendrocyte survival, support for oligodendrocyte progenitor cell (OPC) differentiation, and modulation of astrocyte and microglial activity in a manner that promotes remyelination [24].

Regarding clinical application, four S1P receptor modulators—fingolimod, ozanimod, ponesimod, and siponimod—have been approved by the US Food and Drug Administration (FDA) and the European Medicines Agency (EMA) for the treatment of multiple sclerosis. These drugs are primarily indicated for relapsing forms of MS, including clinically isolated syndrome, relapsing-remitting MS, and active secondary progressive MS. Pivotal Phase III clinical trial data demonstrate that all four agents significantly reduce the annualized relapse rate and decrease the number of gadolinium-enhancing lesions as well as new or enlarging T2 lesions. Some studies also showed a delay in disability progression. Long-term extension studies further confirm that their efficacy can be sustained for up to 5 to 10 years, indicating favorable treatment durability. Notably, siponimod is the first S1P modulator approved for active secondary progressive MS, whereas the efficacy of other drugs in this subtype has not been fully established [25].

Common advantages of this drug class include the convenience of oral administration, sustained long-term efficacy maintenance, and potential direct central nervous system effects. However, their safety profile warrants careful attention. Key concerns encompass cardiovascular adverse effects such as initial dose-related bradycardia, atrioventricular block, and hypertension, with fingolimod exhibiting the most pronounced effects on heart rate. Furthermore, lymphopenia increases the risk of infections, including opportunistic infections such as herpes zoster, progressive multifocal leukoencephalopathy, and cryptococcal meningitis. Other significant adverse events include macular edema, elevated liver enzymes, skin malignancies (e.g., basal cell carcinoma), and decreased pulmonary function (e.g., reduced forced expiratory volume in 1 s (FEV_1_)). Disease rebound following discontinuation, sometimes accompanied by tumefactive demyelinating lesions, can occur with fingolimod, whereas second-generation agents (e.g., ozanimod, ponesimod, siponimod) appear to carry a lower risk in this aspect.

#### 2.1.2. Immunosuppressants

The mechanism of action of immunosuppressants in demyelinating diseases, exemplified by Neuromyelitis Optica Spectrum Disorder (NMOSD), primarily targets its core autoimmune pathology. Based on the pathogenic mechanisms, immunosuppressants inhibit the abnormal immune response through different pathways. Corticosteroids, such as methylprednisolone, as first-line therapeutic agents, exert their anti-inflammatory effects primarily via genomic mechanisms. Lymphocyte proliferation inhibitors, such as Mycophenolate Mofetil (MMF) and Azathioprine (AZA), block T- and B-cell clonal expansion by interfering with purine synthesis. T-cell modulators like Cyclosporine A (CyA) and its analog Tacrolimus both inhibit T-cell activation by suppressing the calcineurin pathway. Among these, Tacrolimus possesses stronger immunosuppressive potency, specifically inhibiting T-lymphocyte proliferation and the transcription of key cytokines such as Interleukin-2. In contrast, Cyclophosphamide (CTX), as a broad-spectrum alkylating agent, non-selectively inhibits lymphocyte proliferation through DNA cross-linking [26].

In terms of clinical application and efficacy, immunosuppressants demonstrate significant differences in their ability to prevent relapses. MMF exhibits the best tolerability and the lowest risk of adverse events (AEs), and its performance in improving the Expanded Disability Status Scale (EDSS) score is superior to that of CTX. Both low-dose CyA and Tacrolimus show therapeutic potential. Clinical observations of Tacrolimus, in particular, indicate that it can promote earlier recovery of neurological function, significantly reduce relapse risk, and demonstrate a pronounced corticosteroid-sparing effect. Conversely, AZA requires 3–6 months of combination therapy with corticosteroids to manifest its effects. Regarding safety, AEs associated with MMF are mostly mild and reversible (e.g., gastrointestinal discomfort), making its safety profile significantly more favorable than those of AZA and CTX. Common adverse effects of Tacrolimus, such as hyperglycemia, tremor, and transient elevation of renal function parameters, require monitoring and its safety profile is generally manageable through dose adjustment, resulting in an overall controllable safety profile.

Nevertheless, immunosuppressants still present safety shortcomings in the treatment of NMOSD. For instance, corticosteroids can cause transient hyperglycemia, and long-term use of cyclophosphamide increases the risk of infection and bone marrow suppression. Additionally, AZA requires concomitant use of steroids, and the long-term safety of MMF (e.g., hypogammaglobulinemia) still requires validation in larger sample sizes. The application of Tacrolimus is currently also primarily based on retrospective studies, and its long-term efficacy and safety need confirmation through more prospective data. Therefore, future efforts should focus on expanding high-quality clinical trials and integrating various therapeutic approaches, including Tacrolimus and emerging biological agents, to further refine personalized treatment strategies [27].

### 2.2. Monoclonal Antibody-Based Therapeutics

The therapeutic role of monoclonal antibodies (mAbs) in chronic autoimmune demyelinating diseases stems from their precise targeting of core immunopathological mechanisms. In neuromyelitis optica spectrum disorder (NMOSD), pathogenesis involves pathogenic anti-aquaporin-4 (AQP4) antibodies mediating astrocyte damage through activation of the complement cascade, antibody-dependent cellular cytotoxicity (ADCC), and inflammatory cytokine release, ultimately leading to blood–brain barrier (BBB) disruption, demyelination, and secondary neuronal injury. Targeting this cascade, mAbs act through distinct strategies: Eculizumab blocks complement protein C5, inhibiting complement-dependent cytotoxicity (CDC) and membrane attack complex (MAC) formation, thereby mitigating AQP4 antibody-mediated astrocyte injury; Satralizumab targets the interleukin-6 receptor (IL-6R), inhibiting IL-6 signaling, reducing plasmablast differentiation and antibody production (including AQP4-IgG), and modulating inflammatory cell infiltration; Inebilizumab and Rituximab target CD19 and CD20 antigens, respectively, depleting B-cell lineages (including plasmablasts and mature B cells) to reduce autoantibody production, with Inebilizumab potentially conferring more comprehensive B-cell depletion due to its broader coverage of subsets, including CD20-negative plasmablasts. Clinically, five pivotal randomized controlled trials (PREVENT, SAkuraSky, SAkuraStar, N-MOmentum, RIN-1) demonstrated significant efficacy; in AQP4-IgG seropositive patients, all treatment groups significantly prolonged the time to first relapse, with Eculizumab achieving a 96-week relapse-free rate of 96%, Inebilizumab reducing relapse risk by 73% at 28 weeks, and Rituximab achieving zero relapses over 72 weeks in RIN-1. Notably, Satralizumab showed no benefit in the AQP4-IgG seronegative subgroup, indicating target-specific efficacy. Regarding safety, overall tolerability is favorable, but Eculizumab requires strict meningococcal infection prophylaxis, and B-cell depleting therapies necessitate monitoring for infection risk and hypogammaglobulinemia. Collectively, these advances highlight the transformative impact of these therapies on NMOSD management, now approved by regulatory agencies in multiple countries; however, challenges persist, including lifelong treatment regimens and high costs limiting accessibility [28,29,30].

In multiple sclerosis (MS), anti-CD20 mAbs (e.g., Rituximab, Ocrelizumab) deplete B cells, reducing antigen presentation and pro-inflammatory cytokine release, thereby indirectly suppressing T-cell-mediated demyelination; Ocrelizumab additionally downregulates cytotoxic function in CD8^+^ T cells and natural killer (NK) cells and reduces Epstein–Barr virus (EBV)-specific T-cell proliferation, potentially enhancing efficacy by limiting heterologous immunity. Natalizumab targets α4β1-integrin, blocking immune cell migration across the BBB and reducing central nervous system (CNS) inflammatory infiltration [31,32]. Clinically, in MS, Rituximab rapidly suppresses new lesion formation in relapsing-remitting MS (RRMS), while Ocrelizumab stabilizes disability progression and reduces serum neurofilament light chain (NfL) levels; however, long-term B-cell depletion may increase infection risk, and Natalizumab requires rigorous monitoring for progressive multifocal leukoencephalopathy (PML).

In summary, mAbs have reshaped the therapeutic landscape of neuroimmune disorders through precise immunomodulation, yet their long-term safety, expansion of indications, and combination strategies require validation in large-scale prospective studies.

### 2.3. Promyelinating and Neuroprotective Agents

#### 2.3.1. Pro-Regenerative Drugs Targeting Oligodendrocyte Maturation

##### GPR17 Antagonists

The mechanism of action of GPR17 antagonists in demyelinating diseases, such as Multiple Sclerosis (MS), is primarily achieved through the regulation of oligodendrocyte differentiation and remyelination. GPR17, an orphan G protein-coupled receptor, is highly expressed in oligodendrocyte precursor cells (OPCs) and pre-oligodendrocytes (pre-OLs). Its activation inhibits adenylate cyclase (AC) via the Gαi signaling pathway, reducing intracellular cyclic adenosine monophosphate (cAMP) levels. This subsequently suppresses the activity of protein kinase A (PKA) and cAMP directly activated exchange protein 1 (EPAC1). This process impedes the differentiation of OPCs into mature, myelin-forming OLs, ultimately inhibiting myelin repair. Antagonists, by blocking GPR17 signaling, relieve this inhibition of the differentiation pathway, thereby promoting OPC differentiation and remyelination [33,34]. Studies have shown that genetic knockout of *Gpr17* or pharmacological inhibition (e.g., using antagonists pranlukast or PTD802) significantly enhances remyelination in animal models (such as experimental autoimmune encephalomyelitis (EAE) [35,36], and the cuprizone or lysolecithin LPC models [37,38]) and improves neurological deficits.

Regarding clinical application, substantial evidence supports targeting GPR17 with pharmacological agents to induce myelin repair, thereby representing a potential disease-course-modifying treatment strategy for MS and potentially other demyelinating diseases. However, the animal models currently used primarily assess the ability of pharmacological interventions to “accelerate” myelin repair, rather than demonstrating their capacity to initiate remyelination de novo; this limits their predictive power for human therapeutics [39]. Furthermore, evaluating therapeutic efficacy in the clinical setting is challenging, as accurately identifying and objectively quantifying the remyelination process in MS patients presents significant difficulties [40]. Even successful remyelination does not halt disease progression in MS animal models, suggesting that promoting remyelination alone may be insufficient as an effective therapeutic strategy. Therefore, combination therapies might be imperative, for instance, co-administering pro-remyelinating agents with approved MS drugs that possess anti-inflammatory and disease-modifying properties.

Currently, two other classes of GPCR antagonists claimed to induce remyelination are under clinical evaluation: the selective M1 muscarinic acetylcholine receptor antagonist PIPE-307 (which blocks Gq/11-coupled M1 receptors) has progressed to Phase II clinical trials for Relapsing-Remitting MS (RRMS); while the LPA1 receptor antagonist PIPE-791 is being evaluated in Phase I trials, intended for the treatment of progressive MS forms, which have thus far proven difficult to intervene against [41].

##### Selective Estrogen Receptor Modulators

Ospemifene, an FDA-approved Selective Estrogen Receptor Modulator (SERM), exerts its core mechanism of action in treating demyelinating diseases by promoting the differentiation and maturation of oligodendrocyte precursor cells (OPCs), thereby enhancing myelination. In in vitro culture systems and on nanofiber scaffolds, this drug notably increases the proportion of mature, myelin basic protein (MBP)-positive oligodendrocytes (OLs), reduces the number of NG2-positive OPCs, and promotes the ensheathment of artificial axonal scaffolds by OLs. In in vivo models, it does not affect OPC proliferation but promotes the transition of OPCs to mature OLs, increasing the myelin area and density of myelinated axons in lesion regions. Concurrently, in the experimental autoimmune encephalomyelitis (EAE) model, it suppresses the overactivation of astrocytes and microglia, alleviating neuroinflammation and creating a favorable microenvironment for remyelination.

Regarding its clinical prospects, given its ability to cross the blood–brain barrier and its safety profile already established in other clinical indications, ospemifene is a candidate for translational research in various myelin injury-related disorders. These include neonatal hypoxia-induced white matter injury (WMI) and demyelinating diseases such as Multiple Sclerosis (MS). It can potentially exert protective effects at the onset of injury and promote myelin repair and neurological functional recovery post-injury. Furthermore, the clear dose-dependent effect observed in EAE models provides a reference for clinical dose optimization.

However, ospemifene has distinct advantages and disadvantages. Its advantages include its status as an approved drug, with established pharmacokinetic data and a low risk of toxicity/side effects. It demonstrates efficacy across different types of myelin injury (e.g., hypoxia-, autoimmune-, and toxin-induced), suggesting broad applicability. The disadvantages include the current lack of clarity regarding its precise molecular target (s)—whether its effects are mediated via estrogen receptors (ERα, ERβ, or GPER1) remains to be confirmed. Clinical application might be associated with adverse effects such as hot flashes. Additionally, its water solubility needs improvement, and precise data on its tissue distribution are still required. These aspects necessitate further investigation and optimization to advance clinical translation [42].

##### Muscarinic Acetylcholine Receptor Antagonists

Clemastine, as a therapeutic agent for demyelinating diseases, primarily exerts its mechanism of action by antagonizing the muscarinic acetylcholine receptor 1 (M1 receptor), thereby promoting the differentiation of endogenous oligodendrocyte precursor cells (OPCs) into mature, myelin-forming oligodendrocytes and stimulating remyelination of demyelinated axons. In the ReBUILD clinical trial, clemastine demonstrated a significant increase in the myelin water fraction (MWF) within the normal-appearing white matter of the corpus callosum in patients with multiple sclerosis (MS). This increase was correlated with a shortening of visual evoked potential (VEP) latency, indicating that it promotes not only structural repair but also functional improvement.

Its advantages include a well-defined promyelinating effect, good tolerability demonstrated in clinical trials, and the feasibility of objective quantification using non-invasive MRI biomarkers such as MWF. However, limitations exist: the efficacy may be region-specific (e.g., pronounced effects in the corpus callosum but not as evident in the corticospinal tract), MWF measurements are susceptible to motion artifacts and noise, and clemastine has not yet been widely adopted in clinical practice. Larger sample trials are still required to further validate its long-term efficacy and safety [43].

##### GABA-A Receptor Modulators

As a positive allosteric modulator specifically targeting GABA_A receptors containing the α3γ1 subunit on oligodendrocytes, β-CCB exerts a demonstrated promyelinating effect in the cuprizone-induced widespread demyelination mouse model. This effect is mediated by enhancing GABAergic signaling and promoting the differentiation of oligodendrocyte precursor cells (OPCs) into mature myelinating cells [44]. Its potential clinical application primarily targets demyelinating diseases such as multiple sclerosis. The advantages of β-CCB lie in its high target specificity, efficacy upon systemic administration without inducing behavioral side effects such as convulsions, and the feasibility of longitudinal treatment monitoring via magnetic resonance imaging. However, this agent also exhibits notable limitations, including suboptimal repair efficacy in gray matter regions such as the cerebral cortex, incomplete concordance between certain imaging metrics and histological outcomes, and an as yet not fully elucidated mechanism underlying the regional heterogeneity of its therapeutic effects. Furthermore, its development remains at the preclinical stage of animal model studies, awaiting further clinical validation.

#### 2.3.2. Epigenetic Modulators

Epigenetic modulators for the treatment of demyelinating diseases have become a focus in neuroscience research in recent years, primarily centered on reversing epigenetic silencing in oligodendrocytes (OLs) and promoting myelin regeneration. The following sections detail these agents according to their mechanisms of action and research progress.

##### HDAC Inhibitors (HDACi)

HDAC inhibitors alleviate neuroinflammation and promote remyelination primarily by modulating microglial phenotype. The HDAC3 inhibitor RGFP966 exerts its effects by suppressing key pro-inflammatory hubs: it downregulates the P2X7 purinergic receptor (P2X7R), thereby inhibiting the subsequent assembly of the NLRP3 inflammasome and the release of IL-1β and IL-18, and concurrently impedes the activation of both the NF-κB and STAT3 signaling axes. This collective action shifts microglia from a pro-inflammatory M1 state to a protective phenotype, reducing oligodendrocyte apoptosis and facilitating remyelination, as demonstrated in cuprizone and experimental autoimmune encephalomyelitis (EAE) models [45,46]. Similarly, other HDAC inhibitors like belinostat show efficacy by targeting overlapping pathways, including the TLR2/MyD88/NF-κB cascade [45].

Regarding clinical potential, studies confirm that RGFP966 significantly improves behavioral deficits and promotes remyelination in demyelination models, suggesting HDAC3 as a potential therapeutic target for neurodegenerative diseases. Its advantage lies in the selective targeting of the HDAC3 isotype, which may reduce the common toxic side effects associated with pan-HDAC inhibitors. However, current research remains limited, as all data originate from preclinical animal and cell models, lacking validation of its safety and efficacy in human trials. As a small-molecule inhibitor, RGFP966 may present risks of off-target effects or unknown toxicity with long-term administration. Moreover, its blood–brain barrier penetration efficiency and in vivo pharmacokinetic properties have not been fully elucidated, necessitating further optimization of dosing strategies. Future work should assess its translational value in primate models and early-phase clinical trials, and explore potential synergistic effects with other anti-inflammatory or pro-remyelinating agents.

##### DNA Demethylating Agents

DNA demethylating agents reactivate silenced genes by reducing promoter methylation. In demyelinating diseases, this primarily aims to reverse the blockade of oligodendrocyte precursor cell (OPC) differentiation.

The nucleoside analogue Decitabine (DAC) has shown promise in the EAE model. Its mechanism is predominantly immunomodulatory; by inducing demethylation of the *Foxp3* enhancer, DAC upregulates *Foxp3* expression, expanding regulatory T cell (Treg) populations. Concurrently, it suppresses pro-inflammatory T-cell differentiation, reducing Th17 cells and related cytokines (e.g., IL-17), which collectively alleviates CNS inflammation [47,48]. Regarding clinical prospects, research confirms that DAC exhibits both preventive and therapeutic effects in the EAE model. Prophylactic administration (initiated pre-immunization or 7 days post-immunization) significantly delays disease onset, reduces cumulative clinical scores, and shortens disease duration, while therapeutic administration (initiated post-onset) also substantially improves symptoms. This supports the rationale for Phase II-III clinical trials of DAC in MS patients. However, the DAC application has notable drawbacks. Firstly, disease relapse was observed after treatment discontinuation, suggesting a need for long-term or maintenance dosing, which raises concerns about genomic instability and potential tumor risk associated with prolonged demethylating agent use. Secondly, while enhancing Tregs, DAC unexpectedly enhanced antigen-specific splenocyte proliferation and significantly increased IL-6 levels upon re-stimulation. This bidirectional immunomodulation could complicate clinical management. Furthermore, DAC’s broad impact on global genomic methylation may disrupt normal gene expression networks, and its lack of specificity risks unforeseen off-target effects [48].

The non-nucleoside inhibitor RG108 promotes remyelination by a more direct route. It blocks DNA methyltransferases, leading to the demethylation and reactivation of key OPC differentiation genes (e.g., *TM7SF2*). This drives OPC maturation via the accumulation of the sterol intermediate FF-MAS, enhancing the expression of myelin proteins. However, current preclinical research for demyelinating diseases remains confined to cell models, lacking support from animal studies or human trial data for its translational potential. A major drawback of RG108 is that its broad suppression of global genomic methylation may lead to excessive activation of tumor suppressor genes and unintended silencing of proto-oncogenes, disrupting epigenetic homeostasis. Furthermore, its aberrant regulation of genes involved in neuronal synaptic plasticity could impair neurological functions such as memory formation.

##### Histone Methylation Modulators

Histone methylation modulators target the epigenetic control of OPC differentiation. The histone methyltransferase EZH2, which catalyzes the repressive H3K27me3 mark, is a key promoter of OPC maturation by silencing inhibitory genes like *SOX6*. In demyelinating diseases, inflammatory microenvironments lead to decreased *EZH2* expression, contributing to remyelination failure [49]. EZH2’s function is intertwined with other modifiers, such as HDACs, suggesting synergy for combination therapies, as seen with dual *DNMT1*/HDAC inhibitors [50].

Regarding clinical applications, gene therapy strategies targeting EZH2 have entered the experimental stage. For example, AAV vector-mediated EZH2 overexpression combined with ciliary neurotrophic factor (AAV-CNTF) significantly promotes remyelination in an optic nerve crush model. The underlying mechanisms include enhanced OPC differentiation and delayed microglia-mediated inflammatory damage. Tailored to different disease stages, this therapeutic regimen can be combined with the leukotriene receptor antagonist Montelukast to promote early OPC differentiation, or with the CSF1R inhibitor PLX3397 to modulate microglial phenotype and support oligodendrocyte maturation.

However, current therapeutic approaches face significant limitations. EZH2 maintains proliferation in neural stem cells but promotes differentiation in OPCs; systemic administration may therefore interfere with normal neural development or potentially induce tumorigenesis [49]. Furthermore, while EZH2-based combination therapies show efficacy in the acute demyelination phase, their effect is limited within the chronic lesion microenvironment characterized by glial scar formation. Finally, the complexity of the epigenetic network renders single-target strategies insufficiently effective. For instance, the synergy between EZH2 and HDAC requires dual-effect inhibitors, yet existing DNMT1/HDAC inhibitors have only been validated in tumor models, lacking data for demyelinating diseases.

#### 2.3.3. Signaling Pathway Inhibitors and Targeted Therapies

##### BTK Inhibitors

Tolebrutinib, an oral Bruton’s tyrosine kinase (BTK) inhibitor with blood–brain barrier penetration, exerts its therapeutic effects in demyelinating diseases through a dual-step mechanism. Firstly, it inhibits the activation of peripheral B cells and myeloid cells, thereby reducing neuroinflammation and their subsequent infiltration into the central nervous system (CNS). Secondly, upon entering the CNS, it modulates the local microenvironment to facilitate the differentiation of oligodendrocyte precursor cells (OPCs) and concurrently protects mature oligodendrocytes, collectively promoting remyelination and preserving existing myelin. This mechanism plays a crucial role in the pathology of non-relapsing secondary progressive multiple sclerosis (SPMS). Regarding clinical application, in the Phase III HERCULES trial, tolebrutinib demonstrated a significant reduction in the risk of disability progression and decreased the number of new or enlarging lesions on MRI, offering a new therapeutic option for non-relapsing SPMS patients who currently lack effective treatments. Its advantages include favorable central nervous system penetration and dual immunomodulatory actions. However, a notable disadvantage is the risk of hepatotoxicity, manifested as elevated alanine aminotransferase (ALT) levels and, in rare instances, severe liver injury requiring transplantation, underscoring the necessity for close monitoring of liver function [51].

##### CCR5 Inhibitors

Thioraviroc is a novel oral small-molecule inhibitor targeting the C-C chemokine receptor type 5 (CCR5). It alleviates neuroinflammation and demyelination by blocking the migration of bone marrow-derived myeloid cells into the CNS. Its potential application in multiple sclerosis (MS) is based on research implicating the CCR5 signaling pathway in MS progression. Preclinical studies have shown that Thioraviroc significantly improves neurological deficits, mitigates myelin loss, and reduces spinal cord immune cell infiltration across acute demyelination, relapsing-remitting, and progressive MS models, demonstrating superior efficacy to the positive control drug teriflunomide. Compared to existing monoclonal antibodies (such as anti-CD20 or anti-IL-6R mAbs), Thioraviroc, as an oral small molecule, offers advantages in administration convenience and potentially lower cost. Its mechanism, focused on inhibiting myeloid cell migration rather than directly targeting B cells or inflammatory cytokines, may complement mAb therapies in combination or sequential treatment strategies. This is particularly relevant for addressing the pathology driven by resident glial cells and peripheral myeloid cells in progressive MS. Regarding safety, Thioraviroc has completed a Phase I clinical trial, demonstrating a favorable safety profile, tolerability, and pharmacokinetic characteristics in healthy volunteers, with no potential Drug–Drug interactions identified. However, its specific efficacy and long-term safety in MS patients require further validation in larger, nationwide multicenter clinical trials. Currently, there are no direct data on the use of Thioraviroc in neuromyelitis optica spectrum disorder (NMOSD), but its CCR5-targeting mechanism suggests potential relevance for similar immune cell migration pathways in NMOSD, meriting further investigation [52].

##### Nogo-A Antagonists

Nogo-A antagonists function by selectively inhibiting the interaction between the Nogo-A protein and its receptors (such as NgR1 and S1PR2), thereby blocking the activation of the downstream RhoA/ROCK signaling pathway. This action mitigates the inhibition of axonal regrowth and oligodendrocyte differentiation, ultimately promoting remyelination and functional neurological recovery.

Regarding clinical application, although preclinical studies in models such as EAE, lysolecithin (LPC)-induced demyelination, and spinal cord injury have demonstrated its potential to improve demyelination, reduce inflammation, and enhance functional recovery, and several Phase I clinical trials (e.g., in multiple sclerosis and amyotrophic lateral sclerosis) have indicated good safety and tolerability, the results of Phase II clinical trials have not met their primary endpoints, leaving its therapeutic efficacy uncertain. The advantages of Nogo-A antagonists include their high target specificity and potential to promote self-repair mechanisms within the central nervous system. The primary drawbacks, however, involve the limited blood–brain barrier penetration of antibody-based biologics, inconsistent clinical efficacy outcomes, and a current lack of sufficient human data to support their broad application [53].

### 2.4. Natural Products from Traditional Chinese Medicine

#### 2.4.1. Gastrodin

Gastrodin has demonstrated significant promyelinating effects in demyelinating diseases, primarily mediated by the targeted activation of the PI3K/AKT/mTOR signaling pathway. In a zebrafish model of CNS development, gastrodin directly binds to the PI3K protein, enhancing its phosphorylation level and subsequently activating downstream AKT and mTOR signaling. This process does not increase the number of oligodendrocytes (OLs) but specifically enhances the myelinating capacity of mature OLs, evidenced by an increased number of myelin segments, expanded cell membranes, and upregulated expression of myelin-related proteins (such as MBP and PLP1). In vitro experiments confirmed the necessity of this pathway, as the PI3K inhibitor LY294002 completely blocked gastrodin-induced MBP expression and OL maturation [54].

Furthermore, gastrodin effectively ameliorated pathology in two distinct demyelination injury models. In the lysolecithin (LPC)-induced mouse model of focal demyelination [55], 14 days of gastrodin treatment resulted in a 40% reduction in lesion volume. Transmission electron microscopy at day 21 revealed decreased g-ratio (indicating increased myelin thickness) and a higher proportion of myelinated axons. Concurrently, the lesion area showed an increase in mature OLs (CC1^+^ cells) and a decrease in oligodendrocyte precursor cells (OPCs, PDGFRα^+^ cells). In the experimental autoimmune encephalomyelitis (EAE) model [56], gastrodin administration, whether initiated at disease onset or peak, delayed the progression of neurological deficits, reduced spinal cord demyelination area by 30%, and promoted the regeneration of CC1^+^ Sox10^+^ mature OLs. Additionally, gastrodin alleviates neuroinflammation by inhibiting the activation of microglia and astrocytes, thereby synergistically improving the lesion microenvironment [54]. As a monomeric compound derived from traditional Chinese medicine, gastrodin shows promise for the treatment of chronic demyelinating diseases like multiple sclerosis (MS), particularly in promoting remyelination.

#### 2.4.2. Astragaloside IV

In the experimental autoimmune encephalomyelitis (EAE) model, Astragaloside IV significantly inhibits the pro-inflammatory M1 polarization of microglia/macrophages, as evidenced by the downregulation of markers including TNF-α, IL-1β, iNOS, and CD16/32, while simultaneously promoting their transition to the anti-inflammatory M2 phenotype, marked by the upregulation of Arg-1, CD206, and IL-10. Furthermore, Astragaloside IV modulates astrocyte polarization, suppressing the neurotoxic A1 phenotype (reducing complement C3 expression) and enhancing the neuroprotective A2 phenotype (increasing S100A10, BDNF, and GDNF expression). Mechanistically, Astragaloside IV acts by inhibiting the TLR4/MyD88/NF-κB signaling pathway, thereby reducing the release of pro-inflammatory cytokines and mitigating CNS inflammatory infiltration and demyelination. Concurrently, it promotes the secretion of neurotrophic factors (such as BDNF and GDNF) from astrocytes, inhibits neuronal apoptosis (reducing cleaved-caspase-3 expression), and enhances oligodendrocyte differentiation and myelin repair capacity, collectively ameliorating EAE pathology [57,58].

As the primary active monomer derived from the traditional Chinese medicine Astragalus membranaceus, Astragaloside IV exhibits therapeutic potential for multiple sclerosis (MS) in preclinical studies. Animal experiments demonstrate that oral administration of Astragaloside IV (200 mg/kg) delays EAE onset, reduces neurological functional scores, attenuates weight loss, and significantly improves spinal cord inflammatory infiltration, demyelination, and neuronal apoptosis. It achieves multi-dimensional neuroprotection by modulating peripheral immunity (e.g., M1/M2 transition of splenic macrophages) and restoring CNS glial cell homeostasis [57].

#### 2.4.3. Triptolide

Triptolide (TP), the core active component of Tripterygium glycosides tablets (TGT), exerts anti-demyelinating effects by targeting the modulation of the PACAP/cAMP signaling axis and its downstream pathways. The mechanism involves multiple facets: firstly, TP significantly upregulates the expression of pituitary adenylate cyclase-activating polypeptide (PACAP), promoting cAMP generation and activating protein kinase A (PKA), thereby regulating downstream signaling. Secondly, TP inhibits NF-κB phosphorylation, reducing pro-inflammatory cytokines (IL-17A, IFN-γ, TNF-α, IL-6) and Th1/Th17-associated transcription factors (*T-bet*, *ROR-γt*). Thirdly, through activation of the PI3K/AKT pathway, TP inhibits oligodendrocyte apoptosis (downregulating *BAX* and upregulating *BCL-2*), alleviating spinal cord demyelination (evidenced by improved myelin integrity in Luxol fast blue (LFB) staining) and inflammatory infiltration (reflected by reduced inflammatory cells in hematoxylin and eosin (HE) staining) [59].

Both TP and TGT containing TP significantly improve neurological functional scores and mitigate weight loss in the EAE model, suggesting their potential as candidate therapeutics for multiple sclerosis (MS). However, the extremely narrow therapeutic window and safety concerns, particularly reproductive toxicity, represent significant challenges for its clinical development.

#### 2.4.4. Tanshinone IIA

Tanshinone IIA (TSIIA) effectively suppresses central inflammatory cascades and halts demyelination pathology in the experimental autoimmune encephalomyelitis (EAE) model through a synergistic mechanism involving multi-target immunomodulation and neuroprotection. TSIIA reduces the infiltration levels of CD4^+^ T cells, CD8^+^ T cells, and macrophages/microglia (Mac-1^+^ cells) within the spinal cord in a dose-dependent manner, with the reduction in CD4^+^ T cells—core effector cells in autoimmune responses—directly correlating with clinical symptom improvement. Concurrently, TSIIA effectively downregulates the expression levels of interleukin IL-23 and IL-17 in brain tissue and serum, thereby blocking the IL-23/IL-17 inflammatory axis. This axis plays a pivotal role in driving Th17 cell differentiation, activating microglia, and inducing oligodendrocyte apoptosis, ultimately mitigating myelin damage. Additionally, TSIIA’s potential antioxidant and neuroprotective properties may synergistically inhibit oxidative stress and excitotoxicity, although this mechanism remains unclear [60,61].

Clinically, TSIIA, as an active constituent of the traditional Chinese herb Salvia miltiorrhiza (Danshen), demonstrates translational potential for multiple sclerosis (MS). Compared to existing immunomodulators with singular mechanisms and significant side effects, TSIIA’s multi-target action (simultaneously suppressing immune cell infiltration and pro-inflammatory cytokine release) exhibits marked advantages in the EAE model: high-dose groups showed a significant delay in disease onset, improvement in neurological functional scores, and mitigation of weight loss compared to controls, with no reported acute toxicity. The established clinical use of its derivatives (e.g., sodium tanshinone IIA sulfonate) in cardiovascular medicine also suggests relatively favorable safety, suggesting its potential as a complementary therapy for relapsing MS, particularly Th17-mediated subtypes.

Clinically, TSIIA, as an active constituent of the traditional Chinese herb Salvia miltiorrhiza (Danshen), demonstrates translational potential for multiple sclerosis (MS). However, its clinical application faces defined limitations, including a reliance on animal model data, unclear precise molecular mechanisms, poor oral bioavailability, and unvalidated long-term safety [60].

#### 2.4.5. Resveratrol

As a natural polyphenolic compound, resveratrol (RSV) significantly downregulates the expression of pro-inflammatory cytokines (such as TGF-β, IFN-γ, IL-1β, IL-6, and IL-17) by inhibiting the activation of the nuclear transcription factor NF-κB, while simultaneously promoting the release of the anti-inflammatory cytokine IL-10. This remodels the immune microenvironment within the central nervous system (CNS). Furthermore, RSV can reduce mitochondrial reactive oxygen species (ROS) via the *SIRT1* pathway, providing neuroprotection independent of its anti-inflammatory effects. In the experimental autoimmune encephalomyelitis (EAE) model, intranasally administered macrophage-derived exosomes loaded with RSV (RSV-Exo) specifically target microglia, suppress their overactivation, reduce demyelination, and promote repair. This process is closely associated with the inherent CNS-targeting capability and blood–brain barrier (BBB) traversing ability of the exosomes [62,63].

RSV holds substantial promise for clinical translation. Its delivery system utilizes macrophage exosomes (RAW-Exo) as carriers. Metabolic labeling confirmed their high affinity for microglia, enabling them to bypass traditional delivery barriers and achieve precise enrichment of RSV within CNS lesions. Intranasal administration further avoids systemic exposure, enhancing local concentrations. In the EAE model, RSV-Exo led to a marked improvement in clinical scores and weight loss, demonstrating superior efficacy compared to free RSV. It concurrently suppressed peripheral and central inflammation, highlighting advantages in multi-system regulation. Regarding safety, no significant toxicity was observed in major organs, and exosomal encapsulation mitigated the inflammatory infiltration in the nasal mucosa caused by free RSV. RSV holds substantial promise for clinical translation, particularly when delivered via engineered exosome systems which enhance its stability and target specificity. Nonetheless, challenges related to its inherent pharmacokinetic properties and the scalable manufacturing of advanced delivery systems remain to be addressed [63].

Although the above-mentioned natural products derived from traditional Chinese medicine (such as gastrodin, astragaloside IV, tripterygium glycoside, tanshinone IIA, and resveratrol) have shown multi-target potential in promoting myelin regeneration and neuroprotection in preclinical studies, their clinical transformation still faces a series of common challenges.

First, the limitations of preclinical models are the primary obstacle. The current evidence is mainly based on animal models such as experimental autoimmune encephalomyelitis (EAE). Although these models can simulate the acute inflammation and demyelination characteristics of human multiple sclerosis (MS), they are difficult to reproduce its chronic progression, heterogeneity and complex glial network. The efficacy of most compounds (such as triptolide and tanshinone IIA) has only been verified in acute-phase models, and their restorative effects on chronic lesions remain unclear, resulting in a significant weakening of preclinical restorative effects in the human body.

Second, pharmacokinetics and blood–brain barrier penetration constitute the core bottlenecks. Most natural ingredients have problems such as low oral utilization, fast metabolism and poor water solubility. For instance, tanshinone IIA and resveratrol, due to their strong liposolubility and low bioavailability, are difficult to achieve effective concentrations in lesions. Although novel delivery systems such as exosomes and nano-micelles offer solutions, they still face challenges such as complex preparation, high cost, and the need to verify batch stability and long-term safety.

Furthermore, standardization and repeatability are also key issues. There are differences in the extraction process, purity and chemical composition of natural products, which may affect the reliability and cross-study comparability of the experimental results. Establishing standardized procedures and consistent experimental protocols for compounds is the foundation for promoting their reliable research and development.

Finally, long-term safety and the narrow treatment window restrict clinical translation. Take triptolide as an example. Although it shows excellent therapeutic effects in the model, its therapeutic window is extremely narrow, and its potential reproductive and hepatotoxicity is significant, which greatly limits its clinical application prospects. Systematic assessment of long-term toxicity is a necessary prerequisite for promoting the development of such active ingredients.

Having reviewed the established and investigational pharmacological agents for demyelinating diseases—from immunomodulators and monoclonal antibodies to pro-remyelinating drugs and natural products—we consolidate their key attributes in Table 1 for a comparative overview.

### 2.5. Novel Therapeutic Strategies and Technologies

#### 2.5.1. Novel Delivery Systems

##### Exosome-Based Combination Therapy

In demyelinating diseases such as multiple sclerosis (MS), exosomes exhibit a dual role. On one hand, exosomes from specific sources can inhibit remyelination; for instance, exosomes released by activated microglia carry *miR-615-5p*, which targets the *MYRF* gene, thereby impeding the differentiation of oligodendrocyte precursor cells (OPCs) into mature oligodendrocytes and consequently suppressing myelin repair. On the other hand, exosomes also have significant pro-repair potential. For example, exosomes derived from the serum of young animals or environmentally enriched animals, as well as those from IFN-γ-stimulated dendritic cells, are enriched with *miR-219* and can effectively promote OPC differentiation and remyelination. Furthermore, mesenchymal stem cell-derived exosomes can exert immunomodulatory and neuroprotective effects by regulating macrophage/microglial polarization, inhibiting inflammatory responses, and promoting the differentiation of regulatory T cells (Tregs). In addition to the above, the potential of exosomes as a delivery platform is being expanded to include key transcription factors. The core myelin repair transcription factor Olig2 can be encapsulated into exosomes, for instance, by genetically engineering HEK293 cells to secrete exosomes carrying the Olig2 protein. These exosomes can be specifically taken up by oligodendrocyte precursor cells, enabling the precise delivery of therapeutic proteins to the lesion site. This Olig2-enriched exosome strategy, as a highly promising cell-free therapeutic approach, offers unique advantages over direct cell transplantation, including lower immunogenicity, stronger targeting capability, and easier storage, thereby providing a new technological direction for remyelination [64].

Although exosomes offer advantages such as high biocompatibility, the ability to cross the blood–brain barrier, and low toxicity, and hold potential as drug delivery vehicles or diagnostic biomarkers, their clinical application still faces challenges. These include heterogeneity in exosome sources and cargo, difficulties in standardizing isolation and purification techniques, and the risk that under certain pathological conditions, they may carry and deliver pro-inflammatory or neurotoxic substances that could exacerbate disease progression. Therefore, future efforts need to further elucidate the functional properties of exosomes from different sources and optimize their targeting capability and safety as therapeutic tools to realize their potential for effective regulation and clinical application in demyelinating diseases [65,66].

##### Nanodrug Delivery Systems

Nanocomposite strategies based on existing drugs show great potential for clinical translation. For instance, the GA-DCL nanocomplex co-encapsulates the first-line immunomodulator glatiramer acetate (GA) and the potent corticosteroid dexamethasone (DCL) within a nanocarrier. This design achieves the dual objectives of synergistic immunomodulation and targeted delivery: GA exerts its foundational therapeutic effect by inducing immune tolerance, while DCL potently suppresses acute inflammatory responses. The introduction of the nanocarrier not only improves drug solubility and stability but also, through surface functionalization (e.g., using ligands targeting brain microvascular endothelial cells), is expected to significantly enhance the efficiency of drug transport across the blood–brain barrier (BBB), leading to greater drug accumulation at CNS lesion sites. Preclinical studies indicate that, compared to combination therapy with free drugs, the GA-DCL nanocomplex more effectively controls neuroinflammation, reduces demyelinating lesions, and demonstrates an improved safety profile in the experimental autoimmune encephalomyelitis (EAE) model. This enhanced safety is primarily attributed to targeted delivery reducing systemic exposure and associated side effects (such as metabolic disorders from long-term DCL use). This strategy provides a new paradigm for innovating the delivery methods of traditional drugs and achieving synergistic enhancement of immunomodulation and neuroprotection [67].

#### 2.5.2. Targeted Antibody Clearance Strategy

##### Extracellular Protein Degradation Technology

The mechanism of action of the Molecular Degrader of Extracellular Proteins (MoDE) platform degraders in demyelinating diseases is primarily based on their bifunctional molecular design: one end specifically binds pathogenic IgG antibodies (e.g., anti-myelin antibodies), while the other end binds the asialoglycoprotein receptor (ASGPR) on hepatocyte surfaces. This facilitates receptor-mediated endocytosis, transporting the target protein to lysosomes for degradation, thereby rapidly clearing autoantibodies and alleviating neuroinflammation and myelin injury [68]. For instance, Bauji Pharma’s KJ103 (a low-immunogenicity IgG-degrading enzyme) significantly mitigates immune attack in Acute Inflammatory Demyelinating Polyneuropathy (AIDP) by degrading IgG antibodies; this drug has received Breakthrough Therapy Designation from China’s NMPA and entered Phase III clinical trials. Meanwhile, Biohaven’s BHV-1300 selectively clears IgG1, IgG2, and IgG4 subtypes (preserving functional IgG3). In a Phase I trial, a single dose (1000 mg subcutaneous injection) reduced IgG levels by 60% with a favorable safety profile. Additionally, Efgartigimod PH20 SC targets the IgG Fc fragment via a similar mechanism and is already approved in the EU for treating Chronic Inflammatory Demyelinating Polyneuropathy (CIDP). Its Phase IV trial (NCT06637072) is currently evaluating the transition from intravenous immunoglobulin (IVIg) to subcutaneous administration. Although MoDE technology still faces challenges regarding central nervous system delivery (requiring blood–brain barrier penetration) and target expansion (currently focused primarily on antibodies), its efficient and selective degradation properties offer a novel strategy for precise immune modulation in demyelinating diseases, with significant progress in clinical translation.

##### Precision Immune Modulators

Neonatal Fc Receptor (FcRn) inhibitors represent a class of precision immune modulators that block the FcRn receptor in endothelial cells, inhibiting IgG recycling. This accelerates the degradation of autoantibodies, reduces serum IgG levels, and mitigates immune-mediated nerve injury. Their mechanism of action differs from the non-selective suppression of traditional immunosuppressants, focusing specifically on the downstream process of “clearing pathogenic antibodies” without directly affecting B-cell counts or complement activity. In clinical application, FcRn inhibitors currently focus primarily on Chronic Inflammatory Demyelinating Polyneuropathy (CIDP). Phase III trials showed that representative drugs like efgartigimod significantly reduce relapse rates and prolong relapse-free survival, with a once-monthly subcutaneous dosing regimen greatly enhancing treatment convenience. In Neuromyelitis Optica Spectrum Disorder (NMOSD), drugs like rozanolixizumab can rapidly reduce anti-AQP4 antibody titers and decrease the risk of acute attacks, with some drugs submitted for relevant indications in Europe and the US, potentially becoming new maintenance therapy options. Their advantages are significant, combining precision and safety by targeting only the metabolic pathway of pathogenic IgGs, avoiding common risks associated with traditional immunosuppressants such as infection, bone marrow suppression, or hepatorenal toxicity. The subcutaneous administration also improves patient compliance, rendering it particularly suitable for patients requiring long-term maintenance therapy. However, notable limitations exist. Their mechanism of action is singular, effective only in antibody-mediated demyelinating diseases, with limited efficacy in T-cell-dominated diseases like Multiple Sclerosis (MS). Furthermore, one trial indicated that some patients experienced rapid and severe clinical deterioration after switching from intravenous immunoglobulin (IVIg) to an FcRn inhibitor, suggesting potential risks during the transition process, possibly related to the sharp decline in IgG levels, IVIg withdrawal effects, or other immune mechanisms. Although FcRn inhibitors offer advantages in dosing convenience and a clear mechanism, their safety in CIDP treatment, transition strategies, and personalized application criteria require further research to optimize clinical use and prevent irreversible neurological damage [69].

#### 2.5.3. Cell Therapies

##### BCMA CAR-T Cell Therapy

B-cell Maturation Antigen (BCMA) CAR-T cell therapy demonstrates a therapeutic role in treating Chronic Inflammatory Demyelinating Polyneuropathy (CIDP), primarily relying on the targeted clearance of plasmablasts and plasma cells expressing B-cell Maturation Antigen (BCMA). This action blocks the production of autoantibodies and their attack on peripheral nerve myelin. The therapy involves a single infusion of genetically engineered CAR-T cells, which expand in vivo and persistently eliminate pathogenic B-cell lineages, achieving a deep reset of the immune system. In clinical application, this study reported two cases of refractory CIDP patients treated with anti-BCMA CAR-T therapy. Both achieved drug-free remission in the short term, with one patient maintaining remission for over 24 months, demonstrating long-term efficacy. The other patient relapsed after 12 months following a severe COVID-19 infection, suggesting that external immune challenges might affect the durability of the response. Regarding safety, both patients experienced only mild cytokine release syndrome, with no severe neurotoxicity or other serious treatment-related adverse events, indicating an acceptable safety profile for this therapy in CIDP [70].

However, this therapy still has certain limitations. Although long-term remission can be achieved in some patients, the risk of relapse persists, potentially associated with the reactivation of pathogenic B-cell clones, reappearance of autoantibodies, and B-cell metabolic reprogramming (e.g., enhanced glycolysis regulated by *RFX5*). Differences in individual immune microenvironments and baseline characteristics may also lead to heterogeneous treatment responses among different patients to CAR-T therapy. Overall, BCMA CAR-T therapy provides a new treatment direction for refractory CIDP. Its mechanism of precisely targeting B cells holds theoretical potential for a curative effect. However, before widespread adoption, further optimization regarding the durability of response, strategies to address relapse, and validation of its efficacy and risks in broader populations are needed.

##### Microglial Replacement Therapy

Microglial replacement therapy, as an emerging cellular intervention strategy, shows unique therapeutic potential in specific types of demyelinating diseases, with its efficacy highly dependent on the underlying etiology and core pathological mechanisms. Research in models of Adult-onset Leukoencephalopathy with Axonal Spheroids and Pigmented Glia (ALSP) has confirmed that a key effect of this therapy is the significant amelioration of myelin pathology. Following transplantation of healthy microglia, abnormal myelination (including both hypermyelination and hypomyelination) present in the model animals was effectively reversed, the G-ratio tended towards normal, and axonal integrity was preserved. This indicates that when microglial dysfunction constitutes the core driver of myelin damage, intervening at the causative level through cell replacement holds fundamental therapeutic value.

Consequently, the application prospects of this therapy can be analyzed broadly for the following two categories of demyelinating diseases: First, for demyelination caused by primary microgliopathies, this therapy may hold curative potential. ALSP itself is a typical example of such diseases, rooted in *CSF1R* gene mutations leading to reduced microglial numbers and functional disturbances, subsequently causing dysmyelination and axonal injury. Similarly, other leukodystrophies caused by specific gene mutations where microglia serve as the core effector cells are, in theory, potential indications for microglial replacement therapy. In such cases, replacing the dysfunctional “root cause” cells can directly halt the myelin destruction driven by their impairment. Second, its application in more common acquired demyelinating diseases, such as Multiple Sclerosis (MS), is more complex but still plausible. The core pathology of MS includes inflammatory demyelination driven by autoimmune T cells and B cells, followed by neurodegeneration. Microglia play a dual role: during acute disease phases, they may adopt a pro-inflammatory phenotype, exacerbating myelin destruction and axonal injury, while in repair phases, they can shift to an anti-inflammatory phenotype, clearing myelin debris and promoting regeneration. Therefore, in theory, microglial replacement could remodel the CNS immune microenvironment. Introducing healthy microglia with anti-inflammatory and reparative properties during a critical window might suppress acute inflammation, protect myelin, and create conditions for endogenous repair mechanisms. However, the challenge lies in the fact that the brain environment in MS is not as naturally conducive to donor cell engraftment as in ALSP, potentially necessitating co-administration of methods like CSF1R inhibitors to “vacate the niche,” which increases the complexity and risks of the treatment regimen.

##### Engineered Mesenchymal Stem Cell Therapy

Beyond replacing diseased cells, empowering stem cells through genetic engineering to enhance their therapeutic properties is another important direction. Among these, the transplantation of Olig2-positive monoclonal mesenchymal stem cells represents an optimized development in cell therapy. These selected or modified mesenchymal stem cells, which highly express the key myelin transcription factor Olig2, not only retain their inherent immunomodulatory and neurotrophic functions but also significantly enhance their ability to drive remyelination. Preclinical animal experiments have shown that the infusion of Olig2^+^ mesenchymal stem cells is significantly more effective than unmodified ordinary MSCs in promoting myelin repair and functional recovery, providing a new optimization pathway for cell therapy strategies [71].

#### 2.5.4. Gene Therapy

Demyelinating Charcot-Marie-Tooth disease (CMT) is primarily caused by mutations in genes expressed in Schwann cells; therefore, gene therapy strategies need to specifically target Schwann cells to restore their normal function. Current research focuses on two representative subtypes—CMT1X (caused by mutations in the *GJB1/Cx32* gene) and CMT4C (caused by mutations in the *SH3TC2* gene)—and employs gene replacement therapy as the core strategy. The mechanism of action involves delivering functionally normal genes into Schwann cells via viral vectors to compensate for the loss of protein function caused by mutations. For instance, in CMT1X, the absence of Cx32 protein leads to dysfunctional gap junction channels within the myelin sheath, impairing the transport of ions and small molecules, which subsequently causes demyelination and axonal degeneration. In CMT4C, the SH3TC2 protein is involved in endosomal trafficking and cell signaling, and its loss results in abnormal myelination and nodal structure.

Regarding clinical application, various delivery routes have been explored to achieve Schwann cell-specific expression, including intrathecal, intraneural, and intramuscular injections. Among these, lumbar intrathecal injection is considered the most promising approach for clinical translation, as it allows viral vectors to diffuse through the cerebrospinal fluid to spinal nerve roots and peripheral nerves, thereby achieving relatively broad biodistribution. Furthermore, to enhance targeting specificity, myelin-specific promoters (e.g., the *Mpz* promoter) can be used to restrict gene expression to Schwann cells, avoiding potential risks associated with ectopic expression. In animal models of CMT1X and CMT4C, gene replacement therapy using lentiviral or adeno-associated virus (AAV) vectors has demonstrated partial phenotypic rescue, including improved myelin structure, increased nerve conduction velocity, and recovery of motor function.

However, this therapy still faces several key challenges. On one hand, some CMT1X mutants (e.g., Golgi-retention mutants) may interfere with the trafficking and function of exogenously delivered normal Cx32, potentially diminishing therapeutic efficacy. This suggests that in certain cases, a combined strategy incorporating allele-specific silencing might be necessary. On the other hand, the transduction efficiency and expression level of current viral vectors in Schwann cells remain limited. While AAV vectors offer advantages such as low immunogenicity and a favorable safety profile, their packaging capacity is constrained, and their tropism for Schwann cells is not yet fully defined. Additionally, the durability of gene expression, along with the biodistribution and safety of these vectors in large animals and humans, requires further validation.

Although no gene therapy for demyelinating CMT has yet entered clinical trials, existing research has laid a crucial foundation for developing targeted therapeutic strategies. Future efforts must deepen in the areas of vector optimization, delivery methods, and targeted regulation to advance these therapies toward clinical translation [72].

#### 2.5.5. Re-Myelination Method Based on CRISPR/Cas9

Research on CRISPR/Cas9-based gene editing for remyelination therapy is currently driving a paradigm shift in the treatment of demyelinating diseases across multiple dimensions. The core value of this technology lies in its ability to enable precise interventions targeting various pathological aspects of myelin injury. In the realm of genetic defect repair, researchers have successfully corrected pathogenic mutations in myelin-related genes, such as *PLP1* and *MPZ*, within oligodendrocytes using homology-directed repair or base editing technologies, thereby restoring their normal myelination function. In the field of cell replacement therapy, induced pluripotent stem cells edited with the CRISPR system can be differentiated into oligodendrocyte precursor cells with myelination capacity. These rigorously quality-controlled cells, upon transplantation, demonstrate excellent migration and differentiation properties in preclinical models, effectively forming myelin structures and significantly improving neurological function. At the level of immune regulation, this technology effectively suppresses aberrant autoimmune attacks against myelin by editing the receptor genes in T cells that recognize myelin proteins. Notably, recent studies have developed intelligent therapeutic systems based on microglia: engineering human iPSC-derived microglia to be activated specifically in demyelinated lesion areas by endogenous promoters like *MX1*, thereby driving the local expression of neurotrophic factors or myelin-degrading enzymes and achieving precise repair within the lesions [73].

Regarding clinical translation, researchers are committed to establishing universal cell therapy platforms. The development of universal oligodendrocyte progenitor cells through knockout of the *HLA* locus offers the potential for scalable treatment. Concurrently, significant progress has been made in developing pathology microenvironment-responsive cell therapies. These engineered cells can sense local inflammatory signals and autonomously initiate repair programs, achieving closed-loop control of the treatment [74]. These strategies are applicable not only to acquired demyelinating diseases like multiple sclerosis but also provide new therapeutic avenues for genetic leukoencephalopathies and neurodegenerative diseases.

Having discussed the above innovative strategies, their key characteristics, current status, and challenges are summarized for comparison in Table 2.

## 3. Challenges and Strategies in Drug Development

### 3.1. Challenges

#### 3.1.1. Disease Heterogeneity and the Lack of Precision Medicine

A core challenge in the field of demyelinating diseases is their significant clinical and molecular heterogeneity, which directly constrains the development of broadly effective therapies and highlights the current deficiency in precision medicine. Different disease types, such as multiple sclerosis (MS), neuromyelitis optica spectrum disorder (NMOSD), and chronic inflammatory demyelinating polyneuropathy (CIDP), possess fundamentally distinct core immunopathological mechanisms. This inherent divergence leads to markedly different drug efficacies; for instance, B-cell-depleting agents like ocrelizumab are effective in MS, whereas complement C5-targeted eculizumab or IL-6 receptor-targeted satralizumab are specifically used for AQP4-IgG-positive NMOSD patients, with the latter showing no benefit in seronegative subgroups. Even within a single disease like MS, heterogeneity exists across different clinical courses (e.g., relapsing-remitting vs. progressive forms) and immune subtypes driven predominantly by Th1, Th17, B cells, or myeloid cells, leading to heterogeneous responses to existing treatments. Presently, beyond a few specific antibodies (e.g., anti-AQP4, anti-MOG), there remains a lack of a robust biomarker system capable of reliably predicting disease progression, treatment response, and remyelination potential. Consequently, the traditional “one-size-fits-all” treatment model fails to meet the needs of all patients. There is an urgent need to advance precision medicine strategies through deepened molecular subtyping and the discovery of novel biomarkers to enable patient stratification and the selection of individualized treatment plans.

#### 3.1.2. The Blood–Brain Barrier Limitation and Central Delivery Hurdles

The presence of the blood–brain barrier (BBB) constitutes a fundamental challenge in drug development for central nervous system (CNS) demyelinating diseases, severely limiting the effective accumulation of therapeutic agents at lesion sites. For large-molecule drugs, such as most therapeutic monoclonal antibodies (e.g., anti-CD20, anti-IL-6R) and even advanced cell therapies like CAR-T, their molecular size and physicochemical properties prevent passive crossing of an intact BBB. This confines their action primarily to the peripheral immune system, resulting in insufficient direct targeting of immune or glial cells within the CNS. This may partially explain why some therapies effectively control peripheral inflammation but have a limited impact on the ongoing neurodegenerative processes within the CNS. In contrast, small-molecule drugs (e.g., S1P receptor modulators, the BTK inhibitor tolebrutinib) are often designed with BBB penetration in mind, enabling direct central actions such as modulating microglia and protecting oligodendrocytes. However, they frequently face issues like uneven brain distribution, poor target specificity, and consequent off-target effect risks. For example, tolebrutinib, while effectively entering the brain, is associated with hepatotoxicity, and fingolimod exerts initial effects on the cardiovascular system. Furthermore, the efficacy of candidate drugs aimed specifically at promoting remyelination (e.g., GPR17 antagonists, the epigenetic modulator RG108) is highly dependent on achieving and maintaining therapeutic concentrations within lesioned areas. Therefore, the BBB delivery barrier not only affects the central efficacy of immunomodulatory drugs but also directly constrains the successful translation of all neuroprotective and pro-repair strategies.

#### 3.1.3. Long-Term Drug Safety and Tolerability Issues

As chronic conditions requiring long-term management, the safety and tolerability of treatments for demyelinating diseases are crucial determinants of the clinical benefit–risk ratio. Numerous core existing therapies are accompanied by characteristic adverse reaction profiles, which raises significant concerns for prolonged use. For instance, S1P receptor modulators (e.g., fingolimod) carry risks of first-dose bradycardia and atrioventricular block, as well as long-term concerns like hypertension, macular edema, and increased infection risk. BTK inhibitors (e.g., tolebrutinib), while demonstrating good central penetration, raise notable hepatotoxicity concerns, with clinical trials observing elevated alanine aminotransferase (ALT) levels and, rarely, severe liver injury requiring transplantation. Furthermore, widely used immunosuppressants (e.g., azathioprine, cyclophosphamide) and B-cell-targeting monoclonal antibodies (e.g., rituximab, inebilizumab), which work by depleting lymphocytes, can lead to hypogammaglobulinemia with long-term or profound B-cell depletion. This impairs humoral immune responses and increases the risk of severe or opportunistic infections (e.g., progressive multifocal leukoencephalopathy). These cumulative toxic risks not only limit the long-term use of many promising drugs but also impose higher demands on treatment monitoring, patient stratification, and individualized risk management strategies.

#### 3.1.4. The Translational Gap Between Preclinical Models and Human Disease

Current drug development for demyelinating diseases is severely hampered by a significant translational gap between preclinical models and human disease. Widely used animal models, such as experimental autoimmune encephalomyelitis (EAE) and cuprizone-induced demyelination, can simulate specific aspects of the disease (e.g., acute inflammation or oligodendrocytotoxicity). However, their highly standardized pathophysiological processes fail to adequately recapitulate the chronic progression, substantial heterogeneity, and complex immune-glial cell interaction networks characteristic of human diseases like MS. For example, the EAE model primarily relies on robust peripheral immune activation, while the cuprizone model lacks an adaptive immune response. This limits the predictive value of these models, as remyelination strategies effective in animals (e.g., GPR17 antagonists, Nogo-A/anti-LINGO-1 antagonists) often face challenges in human trials. A critical case is the failure of the anti-LINGO-1 antibody (opicinumab) in Phase II clinical trials for MS. Despite promising preclinical data demonstrating enhanced OPC differentiation and remyelination in rodent models, the trials did not meet their primary endpoints on clinical disability or remyelination biomarkers. This discrepancy highlights a core issue: animal models primarily assess the ability to “accelerate” repair in a conducive, often young and acutely injured, environment, as opposed to the capacity to initiate regeneration “de novo” within the inhibitory, chronic, and gliotic microenvironment of human MS lesions. The failure of such agents raises significant questions about the long-term neuroprotective benefits of a short-term remyelination stimulus if the underlying inflammatory and neurodegenerative drivers persist. Compounding this problem is the scarcity of sensitive clinical assessment tools. Currently, there is a lack of biomarkers capable of precisely and reproducibly quantifying dynamic myelin changes in vivo. Although MRI techniques like myelin water fraction (MWF) were used to assess clemastine’s efficacy in the ReBUILD trial and as an endpoint in anti-LINGO-1 trials, their measurements are susceptible to artifacts, may show region-specific variability, and their correlation with long-term functional neuroprotection remains unproven, rendering them insufficient as confirmed clinical endpoints presently. This dual limitation—systemic model inadequacy and insufficient assessment tools—constitutes a core bottleneck in translating pro-regenerative therapies from the laboratory to the clinic and critically undermines the reliable prediction of their clinical efficacy and long-term neuroprotective potential.

### 3.2. Strategies

#### 3.2.1. Precision Medicine and Biomarker Development

The core strategy for addressing the heterogeneity of demyelinating diseases lies in advancing precision medicine, which is fundamentally dependent on the development and application of biomarkers capable of guiding patient stratification and therapeutic decision-making. Current successes in targeted therapy, exemplified by the detection of anti-aquaporin-4 (AQP4) antibodies in neuromyelitis optica spectrum disorder (NMOSD), directly determine patient eligibility for treatments targeting complement (e.g., eculizumab) or the IL-6 receptor (e.g., satralizumab), facilitating a transition from “disease diagnosis” to “targeted therapy.”

Future precision medicine must extend beyond single antibodies to integrate multidimensional information, including genetic (e.g., Human Leukocyte Antigen DRB1 (*HLA-DRB1*) allele variants), epigenetic (e.g., DNA methylation patterns of myelin-related genes), and immunophenotypic (e.g., Th1/Th17 bias, B-cell subset distribution) factors. These elements critically contribute to the heterogeneity in drug efficacy and safety profiles across individuals. Emerging therapies—such as BTK inhibitors, GPR17 antagonists, and FcRn inhibitors—are increasingly being evaluated within stratified clinical trials that incorporate such multimodal biomarker profiling. For instance, patient selection based on microglial activation states or oligodendrocyte precursor cell (OPC) differentiation capacity may enhance the success of remyelination therapies. Future clinical development must therefore prioritize the integration of multi-omics data to identify predictive signatures and match patients to optimal therapeutic strategies, thereby advancing truly personalized treatment paradigms in demyelinating diseases.

The development of biomarkers has shifted from single immune indicators to an integrated multi-dimensional evaluation system encompassing imaging, bodily fluids (proteomics/metabolomics), and cellular analyses. This comprehensive approach provides support for precise treatment guidance and early identification of repair responses. Imaging biomarkers have broken through traditional assessment methods: quantitative MRI techniques such as myelin water fraction (MWF) and magnetization transfer ratio (MTR) are already being used to evaluate the efficacy of pro-myelinating drugs; diffusion tensor imaging (DTI) fractional anisotropy (FA) can track axonal integrity; and PET myelin tracers show promise for in vivo quantification of whole-brain myelin content. Among fluid biomarkers, serum neurofilament light chain (NfL), glial fibrillary acidic protein (GFAP), and multiplex protein assays contribute to disease monitoring, chronic injury assessment, and immune subtype classification, respectively. Metabolomics, by analyzing metabolite profiles, can reveal pathway disturbances and distinguish clinical subtypes. At the cellular and molecular level, single-cell RNA sequencing of peripheral blood immune cells enables the dissection of immune status, while circulating oligodendrocyte precursor cell-related miRNAs hold potential as “liquid biopsy” tools for assessing remyelination potential. In the future, integrating multi-omics data and leveraging large-scale biobanks and clinical databases will enable the construction of robust predictive model systems, driving a shift in treatment strategies from “broad-spectrum immunomodulation” toward “personalized neural repair and protection.”

#### 3.2.2. Development and Application of Novel Delivery Systems

Overcoming the blood–brain barrier (BBB) delivery bottleneck is pivotal to unlocking central nervous system (CNS) repair therapies. Novel delivery systems are evolving from “passive penetration” towards “active navigation.” Current research has moved beyond simple chemical drug modifications to focus on developing intelligent, biocompatible carriers. For instance, the GA-DCL nanocomplex, through nano-formulation and functionalization, synergistically enhances the BBB penetration efficiency and lesion targeting of both the immunomodulator and the corticosteroid, achieving dual optimization of efficacy and safety in the experimental autoimmune encephalomyelitis (EAE) model. More instructive are cell-based “Trojan horse” strategies, such as macrophage-derived exosomes loaded with resveratrol, which utilize the innate homing capability of natural carriers to achieve precise targeting of microglia upon intranasal administration, offering a novel approach for the delivery of macromolecules (e.g., oligonucleotides, epigenetic modulators) that are typically difficult to cross the BBB. Looking ahead, the development of delivery systems should become “smarter” and more “integrated”: first, developing intelligent nanomaterials that dynamically respond to the lesion microenvironment (e.g., specific enzymes, pH) for on-demand controlled drug release; second, deeply integrating the delivery system with the therapy itself, for example, designing bifunctional carriers that simultaneously bear targeting ligands (e.g., anti-transferrin receptor antibodies) and pro-remyelinating drugs, or engineering exosomes directly into therapeutic entities with both delivery and immunomodulatory functions. Ultimately, these highly customized delivery platforms will constitute a modular toolbox capable of personalized matching based on drug characteristics and disease stage, thereby potentially enhancing the therapeutic index.

#### 3.2.3. Multi-Target Synergistic Therapeutic Strategies

Given the complexity of the pathological mechanisms in demyelinating diseases, single-target interventions often struggle to curb disease progression throughout its entire course, making the development of multi-target synergistic therapeutic strategies imperative. The core of this strategy is to break away from the traditional linear treatment model of “anti-inflammation first, repair later,” shifting towards the synchronous synergy of immune regulation and neural repair. For example, combining Bruton’s tyrosine kinase (BTK) inhibitors (e.g., tolebrutinib), which possess both peripheral immunosuppressive and direct central microglial modulatory functions, with GPR17 antagonists designed to relieve OPC differentiation blockade, could theoretically create a therapeutic environment where “damage inhibition” and “repair promotion” proceed in parallel. This approach holds promise for substantially promoting remyelination while controlling neuroinflammation, thereby more effectively delaying or even reversing disability progression. A more forward-looking strategy involves developing single chemical entities with multiple pharmacological activities, such as designing bifunctional inhibitors that simultaneously target key epigenetic regulators (e.g., HDAC/DNMT) and specific immune checkpoints, or structurally optimizing S1P receptor modulators to enhance their activating effect on the S1P5 receptor on oligodendrocytes—thereby directly empowering remyelination—while retaining their lymphocyte sequestration function. In the future, rationally designing sequential combination regimens (e.g., initiating rapid immune control during the active disease phase followed by switching to pro-repair maintenance therapy) or multi-functional molecules based on systems biology-driven network pharmacology analysis will be a key path to achieving the paradigm shift from “disease modification” to “functional reconstruction.”

#### 3.2.4. Non-Immune Directed Repair Therapies

Future research and development need to look beyond immunomodulation and focus on strategies that directly enhance the endogenous repair capacity of the central nervous system. This includes using epigenetic modulators to reverse the differentiation block of oligodendrocytes, employing metabolic interventions to reshape the energy metabolism state in lesioned areas, and utilizing small-molecule drugs to induce the polarization of microglia towards a repair phenotype. More innovative directions involve developing curative therapies: employing AAV vector-mediated gene replacement therapy for diseases caused by specific gene mutations (e.g., CMT), and exploring microglial replacement strategies for diseases associated with microglial dysfunction. These non-immune therapies aim to bypass complex immune pathology and directly activate the regenerative potential of neuroglial cells, offering novel pathways for achieving the reversal of neurological function.

#### 3.2.5. Innovation in Clinical Research Paradigms

To address the complex challenges in demyelinating disease research, it is essential to innovate beyond traditional clinical research paradigms. Adaptive clinical trial designs, which pre-specify protocol modification mechanisms (e.g., re-randomizing subjects to more effective treatment arms based on interim analyses), can efficiently evaluate multiple candidate therapies, particularly suited for identifying effective subgroups within highly heterogeneous patient populations. Concurrently, real-world data and digital health technologies should be fully integrated: utilizing electronic health records and registry data to construct external control arms, supplementing randomized trial evidence; quantifying patients’ daily activity levels through wearable devices; and combining novel MRI techniques (e.g., myelin water fraction) with non-invasive biomarkers (e.g., blood neurofilament light chain) to construct a multidimensional efficacy assessment system for the long-term, objective monitoring of neurological function and myelin dynamics. Establishing open, shared real-world data platforms and developing digital twin models that integrate clinical, imaging, and molecular features will significantly enhance the predictive accuracy of clinical trials and accelerate the optimization and validation of individualized treatment plans.

## 4. Conclusions

Research on pharmacological interventions for demyelinating diseases has made significant progress over the past few years, profoundly reshaping the therapeutic landscape for these disabling neurological disorders. The current therapeutic arsenal has evolved beyond early broad-spectrum immunosuppressants (e.g., glucocorticoids and azathioprine) and first-line immunomodulators (e.g., interferon-β and glatiramer acetate), ushering in an era of precision targeting exemplified by monoclonal antibodies (e.g., those targeting CD20, C5, and IL-6R), which have shown considerable efficacy in controlling disease relapse. However, these strategies possess fundamental limitations in reversing neurological disability and promoting remyelination, coupled with challenges such as infection risks and insufficient blood–brain barrier (BBB) penetration upon long-term use. Consequently, the research paradigm is undergoing a profound shift: from “disease modification” centered on inflammation control towards “reparative therapy” aimed at reconstructing neurological function. This shift has spurred the emergence of numerous novel strategies. These include agents designed to relieve the differentiation block of oligodendrocyte precursor cells (OPCs), such as GPR17 antagonists and muscarinic receptor antagonists; epigenetic modulators seeking to reverse epigenetic silencing and restart repair programs by regulating histone modifications (e.g., HDAC inhibitors) and DNA methylation (e.g., DAC and RG108); and novel small molecules with dual peripheral and central actions (e.g., BTK inhibitors and S1P receptor modulators). Furthermore, therapeutic modalities themselves are being revolutionized. Cell therapies (e.g., BCMA CAR-T), extracellular protein degradation technologies (e.g., the MoDE platform), and intelligent delivery systems based on exosomes and nanoparticles collectively represent the next-generation technological platforms poised to overcome current therapeutic bottlenecks.

Looking ahead, several unresolved challenges warrant focused research efforts. A primary hurdle remains the efficient delivery of therapeutics across the blood–brain barrier (BBB), particularly for biologics and remyelination-promoting agents discussed in Section 2.2 and Section 2.3. Future work must prioritize the development of smarter, lesion-targeted delivery systems (as introduced in Section 2.5.1), such as environmentally responsive nanomaterials and engineered exosomes. Secondly, the chronically inflamed, gliotic lesion microenvironment in progressive disease stages presents a formidable barrier to repair, which limits the efficacy of many pro-regenerative agents from Section 2.3. Combating this requires a deeper understanding of the molecular inhibitors present and the development of combinatorial strategies that simultaneously dampen this inhibition (e.g., using microglial modulators from Section 2.3.2 and Section 2.4) and actively stimulate OPC differentiation and maturation. Furthermore, the significant heterogeneity of demyelinating diseases, both between and within diagnoses (a challenge outlined in Section 3.1.1), necessitates a robust precision medicine framework. This relies on the discovery and validation of novel biomarkers—beyond AQP4-IgG—that can predict disease course, treatment response, and remyelination potential, likely derived from multi-omics profiling and advanced neuroimaging.

Specific future directions should therefore include (1) the rational design of multi-target therapies or sequential treatment regimens that synergistically suppress neuroinflammation (leveraging agents from Section 2.1 and Section 2.2) while directly promoting remyelination and neuroprotection (using strategies from Section 2.3 and Section 2.4); (2) intensive investment in non-immune directed repair strategies, such as epigenetic reprogramming (Section 2.3.2), metabolic modulation of glial cells, and cell replacement therapies (Section 2.5.3), to directly address the failure of endogenous repair; and (3) the application of innovative clinical research paradigms (as proposed in Section 3.2.5), such as adaptive platform trials, which are better suited for efficiently evaluating interventions in heterogeneous patient populations and identifying responsive subgroups. By systematically addressing these challenges through interdisciplinary collaboration, the field can accelerate the development of transformative therapies that not only modify the disease course but truly restore neurological function in patients with demyelinating diseases. Ultimately, advancing this systematic endeavor urgently requires innovation in clinical trial paradigms and deep integration of interdisciplinary expertise. By synergistically combining knowledge from genetics, immunology, neuroscience, biomaterials science, and other disciplines, the core challenges—including disease heterogeneity, the translational gap, and long-term drug safety—can be collectively addressed. This concerted effort will propel the ultimate therapeutic goal from delaying disability progression towards the achievement of neurological function restoration.

## Figures and Tables

**Table 1:** Current Clinical Pharmacological Armamentarium for Demyelinating Diseases.

Drug/Therapy Name	Category	Primary Target/Mechanism	Indication (or Research Model)	Stage of Development	Key Features/Challenges	Cited References
Interferon-beta (IFN-β)	Immunomodulator	Inhibits T-cell migration across BBB; downregulates pro-inflammatory cytokines	RRMS, CIS	Approved	First-line injectable; reduces relapse rate; ineffective for PPMS	[15,17]
Glatiramer Acetate (GA)	Immunomodulator	Mimics MBP to induce immune tolerance; promotes Th2/Treg cells	RRMS, CIS	Approved	First-line injectable; delays conversion from CIS to CDMS	[15,16,18,19,20,21,22,23]
Fingolimod, Ozanimod, Ponesimod, Siponimod	S1P Receptor Modulator	Modulates S1P receptors; sequesters lymphocytes in lymph nodes	Relapsing MS forms (CIS, RRMS, active SPMS)	Approved	Oral administration; sustained efficacy; first-dose cardiac effects, infection risk	[24,25]
Mycophenolate Mofetil (MMF)	Immunosuppressant	Inhibits purine synthesis; blocks T/B cell proliferation	NMOSD (Off-label)	Clinical Use/Off-label	Good tolerability; superior EDSS Improvement Vs. CTX; mostly mild AEs	[26,27]
Tacrolimus	Immunosuppressant	Inhibits calcineurin pathway; suppresses T-cell activation	NMOSD (Off-label)	Clinical Use/Off-label	Promotes early neurological recovery; significant corticosteroid-sparing effect	[26,27]
Eculizumab	Monoclonal Antibody	Anti-C5 complement; inhibits CDC and MAC formation	AQP4-IgG+ NMOSD	Approved	High efficacy (96-week relapse-free rate of 96%); requires meningococcal infection prophylaxis	[28,29,30]
Satralizumab	Monoclonal Antibody	Anti-IL-6 receptor; inhibits plasmablast differentiation and antibody production	AQP4-IgG+ NMOSD	Approved	Subcutaneous administration; no benefit in seronegative subgroup	[28,29,30]
Inebilizumab	Monoclonal Antibody	Anti-CD19; depletes B-cell lineages (including plasmablasts)	NMOSD	Approved	Broader B-cell depletion; reduces relapse risk by 73% at 28 weeks	[28,29,30]
Rituximab	Monoclonal Antibody	Anti-CD20; depletes B cells	RRMS (Off-label)	Off-label/Clinical Use	Rapidly suppresses new lesion formation in RRMS	[31,32]
Ocrelizumab	Monoclonal Antibody	Anti-CD20; depletes B cells; downregulates CD8^+^ T cell and NK cell cytotoxicity	RRMS, PPMS	Approved	Stabilizes disability progression; reduces serum NfL levels; long-term B-cell depletion may increase infection risk	[31,32]
Natalizumab	Monoclonal Antibody	Anti-α4β1-integrin; blocks immune cell migration across BBB	RRMS	Approved	Highly effective in reducing relapses and lesions; requires rigorous PML monitoring	[31,32]
GPR17 Antagonists (e.g., Pranlukast, PTD802)	Pro-regenerative Drug	Antagonizes GPR17 (Gi-coupled), relieving inhibition of OPC differentiation and promoting remyelination	MS (EAE, Cuprizone, LPC models)	Preclinical	Promotes remyelination in models; animal models primarily assess “acceleration” of repair, not “de novo” regeneration; clinical assessment challenging	[33,34,35,36,37,38,39,40]
Ospemifene	Pro-regenerative Drug	Selective Estrogen Receptor Modulator (SERM); promotes OPC differentiation and maturation	MS, Neonatal WMI (In vitro, EAE model)	Preclinical/Translational	FDA-approved for other indications; crosses BBB; efficacy in multiple injury models; precise molecular target unclear	[42]
Clemastine	Pro-regenerative Drug	M1 muscarinic receptor antagonist; promotes OPC differentiation and remyelination	MS (ReBUILD Trial)	Phase II Completed	Significantly increased corpus callosum MWF and shortened VEP latency; efficacy may be region-specific	[43]
β-CCB	Pro-regenerative Drug	Positive allosteric modulator of GABA-A receptors (containing α3γ1 subunit) on OLs; promotes OPC differentiation	MS (Cuprizone model)	Preclinical	High target specificity; effective upon systemic administration without inducing convulsions; suboptimal repair efficacy in gray matter	[44]
RGFP966	Epigenetic Modulator	Selective HDAC3 inhibitor; suppresses microglial P2X7R/NLRP3/NF-κB/STAT3 signaling	Demyelination (Cuprizone model, BV2 cells)	Preclinical	Ameliorates demyelination and motor deficits; selective targeting may reduce toxicity vs. pan-HDACi; lacks human trial data	[45,46]
Decitabine (DAC)	Epigenetic Modulator	DNA demethylating agent; upregulates Foxp3, increases Tregs, inhibits Th17	MS (EAE model)	Preclinical	Preventive and therapeutic effects in EAE; disease relapse after discontinuation; long-term use raises concerns about genomic instability and tumor risk	[47,48]
RG108	Epigenetic Modulator	Non-nucleoside DNMT inhibitor; induces genomic DNA demethylation, promotes OPC differentiation	MS (Cell models)	Preclinical	Promotes OPC differentiation; broad suppression of genomic methylation may disrupt epigenetic homeostasis; only cell model data	-
Tolebrutinib	Signaling Pathway Inhibitor	BTK inhibitor (BBB-penetrant); inhibits peripheral B cells & myeloid cells; modulates CNS microenvironment	Non-relapsing SPMS (Phase III HERCULES)	Phase III	Reduces risk of disability progression and new MRI lesions; dual immunomodulatory and central actions; hepatotoxicity risk requires monitoring	[51]
Thioraviroc	Signaling Pathway Inhibitor	CCR5 inhibitor; blocks migration of bone marrow-derived myeloid cells into CNS	MS (EAE models: acute, relapsing, progressive)	Phase I Completed	Oral small molecule; superior to teriflunomide in preclinical models; efficacy and long-term safety in MS patients require larger trials	[52]
Nogo-A Antagonists	Signaling Pathway Inhibitor	Blocks Nogo-A interaction with NgR1/S1PR2; inhibits RhoA/ROCK pathway	MS, ALS (EAE, LPC models)	Phase II (Primary endpoints not met)	Preclinical improvement in demyelination and functional recovery; limited BBB penetration of antibodies; inconsistent clinical efficacy	[53]
Gastrodin	Natural Product	Activates PI3K/AKT/mTOR signaling; enhances myelinating capacity of mature OLs	MS (Zebrafish, LPC, EAE models)	Preclinical	Reduces lesion volume, increases myelin thickness, promotes mature OLs; all data from animal models	[54,55,56]
Astragaloside IV	Natural Product	Inhibits TLR4/MyD88/NF-κB pathway; modulates microglia/macrophage (M1/M2) and astrocyte (A1/A2) polarization	MS (EAE model)	Preclinical	Multi-target synergy (anti-inflammatory, neuroprotective); low toxicity; lacks human trial data	[57,58]
Triptolide (TP)	Natural Product	Modulates PACAP/cAMP axis; inhibits NF-κB; activates PI3K/AKT; inhibits oligodendrocyte apoptosis	MS (EAE model)	Preclinical	Core component of Tripterygium glycosides; improves EAE scores; extremely narrow therapeutic window; reproductive toxicity concerns	[59]
Tanshinone IIA (TSIIA)	Natural Product	Multi-target: reduces T cell infiltration, blocks IL-23/IL-17 axis, potential antioxidant	MS (EAE model)	Preclinical	Multi-target immunomodulation; lipophilicity and low oral bioavailability; lacks human data	[60,61]
Resveratrol (RSV)	Natural Product	Inhibits NF-κB; reduces ROS via SIRT1; (RSV-Exo) delivered via macrophage exosomes for microglia targeting	MS (EAE model)	Preclinical	Exosome delivery enables CNS targeting and avoids systemic exposure; low aqueous solubility and rapid metabolism of free RSV; complex manufacturing of exosomes	[62,63]

**Table 2:** Emerging Therapeutic Strategies in Demyelination Research.

Drug/Therapy Name	Category	Primary Target/Mechanism	Indication (or Research Model)	Stage of Development	Key Features/Challenges	Cited References
Exosome-Based Therapy	Novel Delivery System	Natural carriers for targeted drug delivery (e.g., miR-219, anti-inflammatory agents) to CNS	MS	Preclinical	High biocompatibility, ability to cross BBB; challenges include heterogeneity in sources/cargo and standardization of isolation	[64,65,66]
GA-DCL Nanocomplex	Novel Delivery System	Nano-co-encapsulation of GA and Dexamethasone for synergistic immunomodulation & targeted delivery	MS (EAE model)	Preclinical	Enhances BBB penetration and lesion targeting; improves efficacy and safety vs. free drugs; not yet in clinical trials	[67]
MoDE Platform (e.g., KJ103, BHV-1300)	Extracellular Protein Degrader	Bifunctional molecule binds pathogenic IgG and hepatocyte ASGPR, directing it to lysosomal degradation	AIDP, CIDP (KJ103: Phase III; BHV-1300: Phase I)	Phase I/Phase III	Rapidly clears pathogenic antibodies; KJ103 received Breakthrough Therapy Designation in China; CNS delivery remains a challenge	[68]
FcRn Inhibitors (e.g., Efgartigimod)	Precision Immune Modulator	Blocks FcRn in endothelial cells, inhibiting IgG recycling and accelerating autoantibody degradation	CIDP (Approved in EU), NMOSD	Phase III/Approved (varies)	Subcutaneous administration; precise mechanism; ineffective in T-cell dominated diseases (e.g., MS); risk of deterioration during transition from IVIg	[69]
BCMA CAR-T	Cell Therapy	Targets and clears BCMA+ plasmablasts/plasma cells; blocks autoantibody production	Refractory CIDP (Case Reports)	Early Clinical Research	Achieved drug-free remission (sustained in one patient > 24 months); single infusion; relapse risk exists	[70]
AAV-mediated Gene Replacement	Gene Therapy	Delivers normal genes (e.g., GJB1/Cx32 for CMT1X, SH3TC2 for CMT4C) to Schwann cells via viral vectors	Demyelinating Charcot-Marie-Tooth (CMT)	Preclinical	Partial phenotypic rescue in animal models; challenges include limited transduction efficiency in Schwann cells and durability of gene expression	[72]

## Data Availability

No new data were created or analyzed in this study.
